# Optimizing an evidence-based team-building intervention for dissemination: Collaboration Planning 2.0

**DOI:** 10.1017/cts.2025.10161

**Published:** 2025-09-23

**Authors:** Betsy Rolland, Shruthi Venkatesh, Allan R. Brasier

**Affiliations:** 1 Institute for Clinical and Translational Research, School of Medicine and Public Health, University of Wisconsin-Madison, Madison, WI, USA; 2 Michigan Institute for Clinical & Health Research (MICHR), University of Michigan, Ann Arbor, MI, USA; 3 Department of Internal Medicine, School of Medicine and Public Health, University of Wisconsin-Madison, Madison, WI, USA

**Keywords:** Team science, interventions, team building, program evaluation, dissemination

## Abstract

**Introduction:**

The conduct of Clinical and Translational Research (CTR) requires the engagement of highly effective collaborative teams. Clinical and Translational Science Award hubs have employed team-building strategies to improve team processes and interpersonal relationships in CTR teams. As previously reported, the University of Wisconsin Institute for Clinical and Translational Research (UW-ICTR) team science core operationalized and implemented one such strategy: Collaboration Planning. Here, we report on optimization of that intervention and assessment of three outcomes: (1) Changes in clarity and confidence around team processes; (2) Value and usefulness; and (3) Plans for future behavior change.

**Materials and Methods:**

Collaboration Planning 2.0 improves upon our initial implementation by (1) optimizing the worksheet for flow, accessibility, and deeper discussion; (2) expanding the evaluation process; and (3) creating a facilitator training to support broad dissemination. We tested this iteration in 11 UW-ICTR pilot teams using pre- and post-session self-assessment surveys.

**Results:**

Data indicated an increase in participants’ clarity and confidence around all measured team processes except authorship. Ninety-one percent of participants found the intervention both valuable and useful. Participants indicated plans for future behavior change, including increased attention to team processes. To date, more than 400 individuals have completed the Collaboration Planning Facilitator Training, indicating a deep need in the community for tools for effective team-focused interventions.

**Conclusion:**

These results provide evidence that Collaboration Planning is an effective, accessible, low-barrier intervention for improving team processes and interpersonal relationships in CTR teams. Future work includes expanded evaluation, greater personalization of the intervention, and self-administered facilitation.

## Introduction

The conduct of Clinical and Translational Research (CTR) requires the engagement of highly effective teams working collaboratively across disciplines and across the translational spectrum in pursuit of advancing new treatments or process interventions into practice. However, such research is complex, time consuming, and subject to high rates of failure [1]. One strategy Clinical and Translational Science Award (CTSA) hubs have employed to improve the success of CTR Teams (CTRTs) has been to provide support and facilitation services such as team-building strategies. These strategies, often adapted from those shown effective in corporate or other non-academic environments, are designed to improve team processes and interpersonal relationships in order to drive scientific accomplishments [2]. Yet, few of these strategies have been evaluated for effectiveness in CTRTs and even fewer have been sufficiently tested to support broad dissemination [2]. Rolland and colleagues (2021) detailed “the relative dearth of evidence-based interventions to support team effectiveness” and called for further investment in the development of new and adaptation of existing team-based intervention to the unique context of CTR [3]. Without such targeted interventions, CTSA team science personnel and CTRTs are caught between knowing they need to improve their teamwork and not having access to resources that can help them achieve that goal.

To address this gap, in 2019, the University of Wisconsin-Madison Institute for Clinical and Translational Research (UW-ICTR) team science core, under direction of the first author (BR), began to experiment with creating such an intervention [4–6]. Seeking to move away from didactic team science trainings that were difficult for teams to operationalize and implement, we built a lightweight, evidence-informed team-building intervention. The goal of the intervention was to engage CTRTs in focusing on their team processes in a way that was accessible, active, and actionable.

### Collaboration planning 1.0

We chose to operationalize and implement the Collaboration Planning framework originally developed by Hall, Vogel, and Crowston to enhance team processes [7–9]. Specifically, the overarching goal of a Collaboration Planning session is to help teams establish shared understanding of their approach to collaboration, laying the groundwork for systems of communication and coordination that impact the quality of a team’s interactions and of the team’s science [10].

As previously described, we developed a structured list of questions to be delivered in a 90-minute facilitated session [6]. The questions were designed to help the team think through and decide upon its own unique approach to collaboration. Our intention was to help the team come to a shared agreement on what style of collaboration felt right and appropriate for their unique configuration and context. This co-creation honors the expertise and experience that each team member brings to the collaborative work while acknowledging that agreement upon a shared approach is critical to success.

The output of the Collaboration Planning session was a written document, which we refer to as the team’s “collaboration plan.” At the end of a session, we encouraged the team to spend time completing any questions we had been unable to cover. This written collaboration plan was to be submitted to UW-ICTR pilot program staff. Additionally, the plan could be used as the basis for a multiple PD/PI plan in future NIH submissions.

While CTRTs found the intervention valuable, our observations indicated that some questions were confusing, requiring the facilitator to rephrase. We also found that the topics, which followed the format of the original Hall, Vogel, and Crowston framework, led to repetition. For example, discussion in the “technological readiness” topic, focused on technologies a team would use in its collaborative work, overlapped substantially with the “communication and coordination” section focused on how the team would communicate and coordinate work. In short, the conversation needed more structure. We also noticed that CTRTs left without clarity around next steps. Finally, we wanted to optimize implementation to enhance dissemination to maximize the impact of the intervention. As such, we returned to the drawing board and began working on Collaboration Planning 2.0.

Here, we describe the revised structure of a Collaboration Planning 2.0 session as delivered to 11 teams funded by pilots at UW-ICTR. We also present data on changes in participants’ clarity and confidence around their team’s processes, as well as on their assessment of value and usefulness of the Collaboration Planning sessions and their intention to take action after the session. Finally, we briefly describe our efforts to disseminate Collaboration Planning.

## Materials and methods

### Collaboration planning 2.0: intervention development

**Figure 1.**
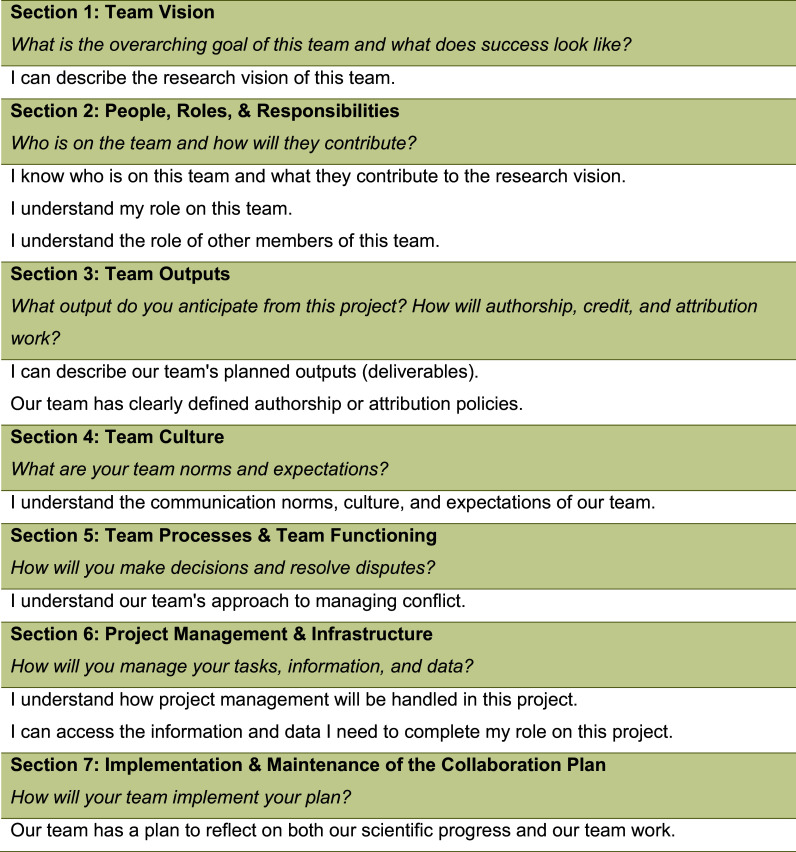
Map of collaboration planning topics to evaluation statements. Response options range from “strongly disagree” to “strongly agree”.

Using data from the original round of testing, we revamped the intervention, which we refer to as “Collaboration Planning 2.0.” (Figure 1 and Supplemental Material Appendix A.) Key changes included (1) optimizing the worksheet for flow, accessibility, and deeper discussion; (2) expanding the evaluation framework and process; and (3) creating a facilitator training and materials to support broad dissemination.

#### Optimizing the worksheet

To address challenges with flow and repetition, we created new categories of questions, starting from the broadest view (the team’s mission and vision), then gradually growing more detailed (e.g., where to store meeting minutes). We also added a section at the end of the session where participants set their intention for how to operationalize the conversation and integrate reflection. In this way, we condensed the ten areas of focus from the original framework to seven sections in Collaboration Planning 2.0. We also transitioned to more open-ended questions that allowed for deeper exploration of topics and simplified language to be more accessible. As such, it is closely related to the coaching approach in which the coach asks so-called “Powerful Questions” to help elicit the beliefs and experience of the participants [11].

#### Expanding the evaluation framework and process

In Collaboration Planning 2.0, we further focused on achieving three key outcomes: (1) Participants felt greater clarity and confidence in their understanding of their team’s approach to collaborative work; (2) Participants felt the intervention was a valuable use of their time and useful enough to recommend to a colleague; and (3) Participants indicated plans for future behavior change.

#### Creating a facilitator training and materials

After the publication of our description of Collaboration Planning [6], we received requests from team science professionals for training in delivering the intervention. In response, Dr Rolland developed a three-hour Collaboration Planning Facilitator Training workshop, with facilitation materials that included the mechanics of delivering a session, background in team science, all session materials (intake form, worksheet, evaluation), and information on developing and evaluating a Collaboration Planning program.

### Recruitment

We offered the opportunity to participate in a Collaboration Planning session to all teams receiving UW-ICTR pilot awards, mechanisms which cover a broad range of research topics, representing basic scientists, clinicians, population scientists, applied researchers, and community-engaged researchers. While the first round of Collaboration Planning described in [6] was presented as a highly recommended, though optional, activity, for the sessions described here, Collaboration Planning was required as a condition of funding.

After receiving funding notices, pilot teams received an invitation from Dr Rolland to participate in a Collaboration Planning session. The invitation described the benefits of Collaboration Planning and contained a link to a brief video about the goals of Collaboration Planning and what the session entailed. Teams were then directed to a registration form (Appendix B) to express interest in participating. PIs were encouraged to invite all team members but given discretion to decide whom that directive included.

### Data collection instruments

Data collection consisted of:

1. *A registration form.* This Google form, completed by the contact PI, requested information about the team, its history of collaboration, and the contact information for team members who should be invited to the session.
2. *A pre-session survey.* Designed to assess each participant’s view of their team’s processes, this REDCap form was emailed to all participants invited to the session approximately two days before the meeting. This survey also collected UW-ICTR’s standard demographic data and asked for each participant’s goals for the session.
3. *A post-session survey.* Distributed to everyone invited to the session immediately after its conclusion, this survey repeated the team process assessment statements. It also requested feedback on the session itself and how participants would apply what they learned.

All data collection instruments are available in the Supplementary Materials (Appendix B).

Responses to team process statements (e.g., “I can describe the research vision of this team.”) were measured using standard 5-point Likert scales (e.g., from “Strongly disagree” to “Strongly agree”). These statements emerged from the worksheet topics. For example, the goal of “Section 1: Team Vision” is for team members to be aligned around what the team is trying to achieve and what success looks like. Thus, the team process statement for assessing that goal is “I can describe the research vision of this team” (Figure 1). We repeated these measures in the post-session survey to assess change.

### Data analysis

We conducted descriptive analyses of demographics, team process responses before and after the Collaboration Planning session, and session feedback questions. We also present overall average scores for the evaluation and team process questions. Because these data did not meet normality assumptions, we used the Wilcoxon Signed Rank Test to examine pre- and post-session team process scores, accepting a *p* < 0.05 as significant.

Open-ended qualitative responses in the surveys were analyzed using thematic analysis. Each response was reviewed by both the first and second authors, and consensus on the themes was reached through discussion and comparison of responses. Responses could be assigned to more than one theme.

This work was deemed program evaluation or “not human subjects research” by the UW-Madison IRB.

## Results

### Participants

Between August 2022 and April 2023, 11 UW-ICTR pilot awardee teams participated in Collaboration Planning sessions facilitated by Dr Rolland. Across those teams, 77 team members were invited to participate in a session; 68 attended (Table S1). Of those 77 invited team members, 69% (*n* = 53) completed the pre-session survey, while 58% (*n* = 45) completed the post-session survey, and 53% (*n* = 41) participants completed both.

Nearly half of the pre-session survey respondents (49%) were from the School of Medicine and Public Health, while 21% were from the College of Pharmacy. Additional participants represented other UW schools and colleges, the UW health system, other research institutions outside of UW, or community partners. Our sample identified themselves as 75% White, 17% Asian, and 92% Non-Hispanic. Our sample was 66% female, 26% male and 4% nonbinary/gender nonconforming (Table 1).

**Table 1.** Participant demographics, *n* = 53

	# (%)
**School/College of Primary Appointment**	
College of Agricultural & Life Sciences	2 (4%)
College of Engineering	1 (2%)
College of Letters & Science	2 (4%)
School of Pharmacy	11 (21%)
School of Medicine & Public Health	26 (49%)
Other (including community partners, UW Health and UW Medical Foundation)	8 (15%)
Did not respond	3 (6%)
**Race and Ethnicity**	
Not Hispanic or Latino origin	49 (92%)
Hispanic or Latino origin	2 (4%)
Did not respond	2 (4%)
White	40 (75%)
Asian	9 (17%)
Other	2 (4%)
Did not respond	2 (4%)
**Gender**	
Female	35 (66%)
Male	14 (26%)
Nonbinary/gender nonconforming	2 (4%)
Prefer not to answer	2 (4%)

The registration form, completed by the contact PI, requested information on the team’s collaboration history (Table S2). Nine of the 11 teams completed this registration survey, while the remaining two teams scheduled the Collaboration Planning session directly without completing the registration. Of these nine teams, six selected both “Members of our team HAVE collaborated previously” and “Members on our team have NOT collaborated previously.” indicating that at least some new team members were being integrated into an existing collaboration. Eight of the nine teams considered themselves interdisciplinary, defined in the survey as engaging multiple aspects of the translational research spectrum, while six teams would be working with community partners.

### Participant assessment of value and usefulness

Overall, participants expressed strong satisfaction with their participation in a Collaboration Planning session. Of the 45 participants who completed the post-session survey, 41 (91%) agreed or strongly agreed that the session was valuable, *Mean*_*valuable*_ = 4.4 [1 = strongly disagree; 5 = strongly agree]. When asked if they would recommend Collaboration Planning to a colleague, 91% indicated they were likely or very likely to do so, *Mean*_*recommend*_ = 4.5. Finally, when asked about the effectiveness of the facilitator of the session (BR), 98% indicated the facilitator was effective or very effective, *Mean*_*effective*_ = 4.6 (Table S3).

### Participant team process responses

**Figure 2.**
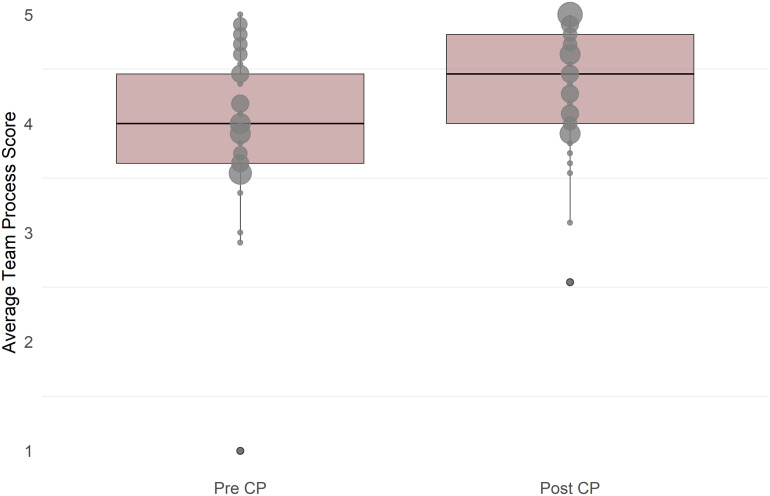
Average pre-post team process score. Boxplots highlight the score distribution (median, interquartile range, and range). Circle size indicates the number of participants with the same score.

We analyzed pre- and post-session survey responses on team processes for the 41 participants who completed both assessments. Due to violation in the normality assumption, a Wilcoxon signed-rank test was conducted to examine overall changes in participants’ team process responses. There was a significant increase in scores from pre- (*Mean* = 4.00, *SD* = 0.71, *α* = .95) to post-session (*Mean* = 4.34, *SD* = 0.56, *α* = .93), *V* = 70, *p* < 0.001 (Figure 2). Descriptively, the Collaboration Planning session enhanced team members’ clarity around roles and collaboration processes, as evidenced by higher proportions of “Strongly Agree” responses post-session across almost all items (Table 2). The exception was the statement, “Our team has clearly defined authorship or attribution policies,” which had minimal change in participant scores.

**Table 2.** Survey results: percentages of responses on the 1-5 point scale before and after the collaboration planning session

	Strongly Agree		Agree		Neutral		Disagree		Strongly Disagree	
Survey item	Pre	Post	Pre	Post	Pre	Post	Pre	Post	Pre	Post
I can describe the research vision of this team.	44	68	49	29	5	2	–	–	2	–
I know who is on this team and what they contribute to the research vision.	41	68	46	29	10	2	–	–	2	–
I understand my role on this team.	44	63	51	32	2	2	–	2	2	–
I understand the role of other members of this team.	39	63	49	32	10	2	–	2	2	–
I can describe our team’s planned outputs (deliverables).	27	51	63	41	7	5	–	2	2	–
Our team has clearly defined authorship or attribution policies.	17	17	34	34	37	41	10	7	2	–
I understand the communication norms, culture, and expectations of our team.	32	49	46	44	15	7	5	–	2	–
I understand our team’s approach to managing conflict.	17	32	39	44	32	20	10	5	2	–
I understand how project management will be handled in this project.	15	46	59	41	22	10	2	2	2	–
I can access the information and data I need to complete my role on this project.	34	51	46	34	15	7	2	7	2	–
Our team has a plan to reflect on both our scientific progress and our teamwork.	20	39	46	46	24	12	7	2	2	–

Notably, individual-level data indicate that a majority of our participants did not change in their average score for each item from the pre-session to post-session survey (Tables S4 and S5). Upon further examination, we see that in the pre-survey, most team process items showed high baseline agreement, with 66% or more participants selecting “Agree” or “Strongly Agree.” The exceptions were the items on authorship (51%) and conflict (56%), which had comparatively lower initial agreement levels.

### Impact of collaboration planning topics

Participants indicated which 2–3 topics of the worksheet they believed would have the most impact on their team’s collaboration (Table 3). The modal topic was Section 5: Team Processes & Team Functioning (63%), where the team discusses decision-making and conflict resolution. The second most selected was Section 2: People, Roles, & Responsibilities (51%), while the third was Section 6: Project Management & Infrastructure (41%).

**Table 3.** Collaboration planning worksheet impact

Which sections of the Collaboration Planning Worksheet do you think will have the most impact on your team’s collaboration practices? (Please select your top 2-3.)	# (%)
Section 5: Team Processes & Team Functioning	26 (63%)
Section 2: People, Roles, & Responsibilities	21 (51%)
Section 6: Project Management & Infrastructure	17 (41%)
Section 3: Team Outputs	14 (34%)
Section 7: Implementation & Maintenance of the Collaboration Plan	14 (34%)
Section 4: Team Culture	10 (24%)
Section 1: Team Vision	7 (17%)

### Open-ended survey questions

The surveys included three open-ended questions: (1) “What are your goals for participating in this Collaboration Planning session?”; (2) “What is one action you will take as a result of participating in this session?”; and (3) “What suggestions do you have for the session facilitator for improving the collaboration planning service? Are there things you would like to see changed or added? If so, what?” The first question was fielded in the pre-session survey while the second and third were fielded in the post-session survey.

#### Pre-session survey

Of 42 responses to the question on session goals (Table 4), the most common (14 responses, 33%) was to enhance collaboration within their team (“Set up mechanisms for productive work throughout the year” and “building relationship with partners in WI who care about the well-being of rural communities”). The second most common goal was a desire to learn team science best practices (13 responses, 31%, “To learn more skills and approaches to team science”).

**Table 4.** Open-ended responses

What are your goals for participating in this Collaboration Planning session?		
Enhance team collaboration (33%)		“Enhance team dynamics and formalize project management.”
		“Be an effective project team”
Learn team science best practices (31%)		“Learn effective and efficient ways to collaborate”
		“Learn methods and ways to best work in a collaborative team, understanding important aspects of collaborations to determine at the beginning of projects, how best to mange team dynamics and any other topics you all feel are important to address based on previous experiences working with other teams.”
Gain project clarity (31%)		“To further describe our project, goals, and roles.”
	Clarity on own role within project	“Better understanding my role/expectations for this project”
Better understand team members (14%)		“I would love to learn new ways to establish and provide opportunities for growth to all team members.
Future proposals (10%)		“To review our project goals and data collection plan that help guide us for our next research study. Also, to discuss the best course of action on future grant opportunities.”
New member onboarding (9%)		“Learn about the new members of the team”
Leadership and mentoring (7%)		“To become more deliberate in planning for and implementing this project.”

What is one action you will take as a result of participating in this session?		
Create written documentation (60%)		“Careful documentation of the course of the project to assist with project write-up in the future.”
	Authorship policy	“Determine how authorship will be decided”
	Written collaboration plan	“Developing a collaboration plan and a plan for reflection”
Clarifying team processes (27%)	Role, task, norm clarity	“Deciding on a data storage and sharing process”
	Goal and vision clarity	“Go over vision and plan recap at regular team meetings.”
Meetings (10%)		“Attend quarterly team meetings”
Relationships (5%)		“Find time to work with partners absent at the session and work on the collaboration planning.”

What suggestions do you have for the session facilitator for improving the Collaboration Planning service? Are there things you would like to see changed or added?		
Personalization (40%)		“Ask team members to highlight specific sections they would like the team to discuss further”
Examples of effective plans (20%)		“Sharing more details on how others have been documenting their collaboration plan; what has seemed successful yet efficient”
Facilitation (13%)		“Facilitation is incredibly important when engaging a group in collaborative planning. The facilitator was mostly just reading from the Google doc and felt short, almost militant, and rushed.”
Reminder about notetaking (13%)		“It would have been good to take notes during the meeting. Maybe the facilitator should encourage participants to do so.”

A third theme was gaining clarity around the project (13 responses, 31%), such as team roles, deliverables, or communication (“Identifying buy-in and deliverables for each team member and a timeline for each”). Three participants (7%) wanted greater clarity about their own role in the project (“Better understanding (of) my role/expectations for this project”).

Some participants (6 responses, 14%) aimed to better understand their team members’ working styles and professional goals to support mutual growth (“I want to expand my understanding of how my colleagues like to work and how we can match each other’s needs, strengths to surpass weaknesses or challenges we face”). Others were looking ahead, hoping the session would help them prepare for future grants (4 responses, 10%, “I would like to prepare our team for submitting either an R21 or R01 this fall”).

#### Post-session survey: action item

In the post-session survey, participants were asked to identify one action they would take after the session. Of the 37 responses, 22 (60%) indicated they would create some form of written documentation for their team (e.g., “Make sure I am clearly documenting my work on the project … in our shared Box or Google Docs so it is available to all team members”). This category included creating an authorship policy (*n* = 10) and updating their written collaboration plan (*n* = 8).

Another common theme was clarifying team processes (10 responses, 27%). This clarification intent included defining roles, tasks, communication norms (*n* = 7, “I will start more clearly defining people’s roles and regularly enforcing our team’s expectations”) or team vision and goals (*n* = 3, “Go over vision and plan recap at regular team meetings”).

Four participants (11%) noted plans to improve meetings (“Incorporate check-in times with the team”), while two (5%) planned to hire additional team members (“Bring in additional personnel to ensure the investigative team is supported”). Another two (5%) mentioned working on relationship-building with collaborators and community partners (“I will need to take a stronger role in trying to facilitate community partner openness”).

#### Post-session survey: session feedback

Fifteen participants provided feedback on the Collaboration Planning session and facilitation. The most common suggestion (6 responses, 40%) was to personalize the session to the team (“Perhaps individualize in advance a bit more for teams that have been working together a while”). Three requested examples of successful collaboration plans (20%, “Sharing more details on how others have been documenting their collaboration plan”).

A couple participants expressed a desire for more interactive facilitation (13%, “Maybe prompt individuals to respond (or start) when introducing a new topic”) and two others recommended reminding teams to take notes during the session (13%, “It would have been good to take notes during the meeting. Maybe the facilitator should encourage participants to do so”). (NB: Each session’s introduction included several nudges to participants to take notes in the provided shared Google doc.)

Three responses (20%) were explicitly positive (“I think the discussion is very interactive and fruitful. As the PI, I have learned a lot such as how to improve management skills for a project that multiple labs involve”).

### Dissemination

As mentioned above, one of our objectives in the Collaboration Planning 2.0 redesign was to create a facilitator training and materials to support broad dissemination. To date, the Collaboration Planning Facilitator Training has been delivered to over 400 facilitators through organizations such as the International Network for the Science of Team Science (INSciTS), the National Organization of Research Development Professionals, and the CTSA-based Team Science Affinity Group (now operating as the ACTS Team Science Professionals Special Interest Group), as well as through a free virtual course hosted on Dr Rolland’s website (www.TheTeamScienceLab.com). A combined 120 individuals participated in the two INSciTS trainings (2021 and 2022), with 38 (32%) completing post-session evaluation surveys. Combined evaluation data from these two trainings (the two for which we can report consistent evaluation data) indicate that 100% of evaluation respondents were satisfied or very satisfied with the training (*Mean* = 4.9/5.0), 100% agreed or strongly agreed that they would recommend it to a colleague (*Mean* = 4.8/5.0), and 92% agreed or strongly agreed that they felt confident facilitating a Collaboration Planning session (*Mean* = 4.3/5.0), indicating enhanced efficacy of the training.

## Discussion

Here, we have described the revision, testing, evaluation, and initial dissemination of a team-building exercise tailored for CTRTs. We sought to further refine our implementation of an evidence-based framework to facilitate teams’ development and understanding of their shared norms and vision. We have drawn upon the science of team building, which can be first traced to the corporate world, where it focused on goal setting and task accomplishment [12] (Reviewed in [2]). However, these early team-building interventions have had only moderate impact on team performance in CTR due to differences in goals, organizational factors, and team characteristics [13]. CTRTs are unique in their composition, environment, and organization and, thus, require an intervention that has been tailored to and evaluated with these unique characteristics in mind. Our data show that Collaboration Planning is an intervention that is both effective and valuable for CTRTs in the academic setting. Moreover, we interpret the high level of interest shown in the CP training as indication of a deep need in the community for tools for effective team-focused interventions.

In this implementation and evaluation of Collaboration Planning, we saw evidence that the intervention is achieving its desired outcomes, which included (1) greater clarity and confidence around team processes, (2) value and usefulness, and (3) plans for future behavior change. Further, Collaboration Planning provides a structured approach to talking about collaborative work that does not require participants to become experts in the Science of Team Science while being a relatively lightweight intervention to implement.

Before the session, participants indicated they were looking for clarity on team processes and for behavior change at both the individual and team levels. Post-session data indicate that, indeed, participants planned to take actions that would further clarify a team process (role, responsibilities, authorship) and/or seek to change the team’s behavior (schedule recurrent meetings). Our results demonstrated:

### Increased clarity and confidence

Overall, participants’ understanding of team processes significantly increased after the Collaboration Planning session. This change was reflected across almost all survey items. We find the stagnation in clarity and confidence in the category of authorship and attribution especially interesting. Our experience in delivering Collaboration Planning is that most participants come into the session agreeing that they understand their team’s authorship policies. Yet, after discussion and digging into the details of said policies, nearly 30% of participants felt less sure. It is quite possible that, through our discussions, participants came to understand that team processes are more nuanced and complex than they had previously believed. This is likely true for authorship and attribution but perhaps applies to other topics as well.

### Value and usefulness

Nearly all participants found their participation in Collaboration Planning to have been valuable and useful, as indicated by their willingness to recommend it to a colleague. We believe that this finding may be one of our most important, as historically, we have found it challenging to engage teams in “meta-scientific” work designed to improve their team processes. Indeed, looking back at the first round of Collaboration Planning done at UW-ICTR, only 13 of 36 teams invited to participate did so.

This finding is especially interesting in light of the large portion of participants whose evaluation scores stayed the same for most topics. Herein, it is conceivable that participants overestimated their initial understanding of team processes at baseline. Having reached the ceiling of the scale, they had limited room for improvement, even if they had learned something during the CP session. We believe this potential contradiction is worth further exploration. If individual participants were not increasing their clarity and confidence, what specifically made their Collaboration Planning participation so valuable and useful? Was it simply enjoyable to engage in conversations about teamwork? Or was there an (as-yet) invisible change in the team’s collaborative spirit that we have not captured?

### Plans for future behavior change

Participants described actions they intended to take to improve their team’s approach to collaboration, including creating written documentation, clarifying team processes, and developing relationships. (Table 4.) In essence, participants described their intention to pay more attention to how their team worked together. It is this increased and sustained attention to team processes that we believe has been missing from team science projects.

### Facilitation reflections

While we did not have a formal process for compiling observations about each session, here we share several anecdotal facilitation reflections. First, while we aim to cover all the worksheet questions in 90 minutes, this is rarely possible. As such, one of the jobs of the facilitator is to get a sense for where the team is in its collaborative processes and steer the team toward topics that may be most useful. Occasionally the team may find itself focusing a disproportionate amount of time on a specific topic or question; this has been especially true for authorship policy. Whether this is because the question can be sensitive – after all, publications are the currency of academia – or because everyone thinks their approach is the “correct” one is an open question.

Second, as alluded to, participants seemed reluctant to take notes in the online document. Whether to have a member of the facilitation team take notes on behalf of the team is a philosophical issue we have explored. We believe that taking notes is an essential part of the co-creation process for the team. Further, we believe that the answers to the question must be in the team’s own language to avoid our expertise being substituted for their decisions. It is easy for the notetaker to convert the team’s words into their own preferred language (in our case, Science of Team Science language), which takes away the team’s agency in making decisions.

Third, several questions challenged most, if not all, teams:

- How can your team create a shared vision of what success looks like for the project as a whole and the individual components? (e.g., kick-off meetings, highlighting the vision at each meeting)
- What are some of your team norms and expectations? How would you describe your team’s culture to a new person? (e.g., we value autonomy and collaboration equally; when something doesn’t go as planned, we regroup and decide next steps together; we set aside time for getting to know each other and have fun; we believe good ideas and leadership can come from anywhere on the team)
- How can you make that team culture explicit and communicate and enforce those team norms and expectations for both existing and new team members?
- What is your process for making decisions?
- What is your process for resolving disputes such as those over resources or deliverables? Consider different kinds of conflict, including interpersonal conflict vs scientific conflict and conflicts among staff vs conflicts between PIs.

We believe that these are aspects of collaborative work that are implicit in how a PI leads a team but are rarely explicitly discussed. As such, few teams had language to describe their approaches to things like creating a shared vision, team norms, decision-making, or resolving disputes. To restate this point, the teams were engaged in these activities but were unsure about how to talk about them.

Finally, teams frequently ask for an example of a “good” or “successful” written collaboration plan, which we do not provide. The reason for that is twofold. First, we have not yet done the research to assess how teams are using their written collaboration plans and whether those plans have an impact, making it impossible to develop criteria (objective or subjective) for what might constitute a good plan. Second, we are concerned that, given an example, teams will see that sample’s content as *the right answer* and adjust their team’s processes or their own plan text to match that.

### Limitations

There are aspects of this work that may limit our ability to generalize these results to science teams more broadly. First, our participants and facilitation team may not be representative of the broader scientific community. Sessions took place at one R1 university, with a large CTSA and team science program with deep expertise in both team science and facilitation. Second, teams were pilot teams who had received funding to support their collaborative work. We worked with these teams in a specific context within a specific moment in time of their team development. They had already – at least some portion – come together around a research idea and had been successful in securing funding. Third, we did not collect data on role or career stage, so we do not know whether those variables might have impacted participants’ self-assessments. Fourth, our results are indicative of the thoughts of a subset of participants and may not represent the experience of all session participants.

The process of Collaboration Planning has its own limitations. First, it is impossible to cover all important aspects of teamwork thoroughly in a 90-minute session. In designing the intervention, we focused our questions on what we believe (and the literature has shown) to be important elements; however, every team may not need or benefit from every question. Other facilitators may want to add other topics, such as questions about community engagement or knowledge integration. Second, as mentioned above, we have limited data on the written collaboration plan and its impact on collaborative work. Finally, our evaluation collects only self-assessment data without any objective measures of improved teamwork. Further, those evaluation data are collected at one moment in time, without any data on mid- or long-term outcomes.

### Future directions

As we consider the future of Collaboration Planning, the intervention itself, as well as its continued implementation and dissemination, we plan to focus on the following enhancements, led by Dr Rolland, now with the Michigan Institute for Clinical and Health Research (MICHR).

#### Evaluation

We plan to build a broader and deeper evaluation that moves beyond self-assessment of clarity and confidence, satisfaction, and stated behavior change to include mid- and long-term outcomes such as psychological safety, achievement of specific aims, or sustained attention to team processes. We are investigating ways of following up with teams at various time points after a session to understand how the discussion impacted them and their collaborative work.

#### Greater personalization

Each team has its own history, its own mix of personalities, goals, and disciplines, and its own trajectory. To date, Collaboration Planning has been a one-size-fits-all intervention, designed for relatively early pilot teams, albeit with content that can help nearly any team. While the intervention lends itself to adaptation, such adaptation relies heavily on the skills of the facilitator. As we move into the next phase of development, we have started testing versions to personalize the worksheet for different team stages.

#### Facilitation options

While we believe strongly in the power of neutral, caring facilitation as an element of Collaboration Planning, we also understand that not every team has access to that kind of support and not every team is ready to engage in full-scale Collaboration Planning. As such, we are developing alternatives to traditional facilitation, including self-guided sessions.

#### Evaluation of written collaboration plans

We need to understand more about what makes for a good written collaboration plan, how teams use them, and how the content of a team’s written collaboration plan may influence their scientific outcomes. As we deliver future Collaboration Planning sessions at MICHR, we plan to collect draft collaboration plans, give feedback, then collect their final version as part of the mid- and long-term evaluation described above.

#### Continued dissemination

In the future, we hope to follow up with those ∼400 trained Collaboration Planning facilitators to better understand their experience facilitating Collaboration Planning, as well as to identify how they have adapted the intervention to their local contexts. As we continue to enhance the existing version, we will be increasing our dissemination activities.

## Conclusion

We have shown here that Collaboration Planning is an effective Team Science intervention that is both valuable and useful to CTRTs in focusing their attention on their teamwork. And while we believe that Collaboration Planning is a useful tool for facilitating deeper conversations about teamwork, collaborative approaches, and team processes, we also believe that the real magic of Collaboration Planning is in engaging a team in a respectful, practical conversation about their collaborative work that results in tangible intent to take action. When teams spend time listening to one another’s ideas about and experience with collaboration, they begin to see the potential for deeper relationships and deeper trust, critical factors in collaborative success.

As of this writing, Dr Rolland has conducted Collaboration Planning session with more than 70 teams, at UW-ICTR and MICHR, as well as across the US. While there have occasionally been contentious questions or topics, she has not experienced any team that was so out of synch that they were unable to have respectful, productive, constructive discussions about making their teamwork more efficient and effective. In fact, we have seen many Collaboration Planning sessions serve as opportunities for team members to express gratitude for one another. This capacity for open conversations is indicative of the dedication to their craft and an understanding of the importance of their shared scientific and health improvement goals that we see in most science teams. Watching teams, in just 90 minutes, progress from occasionally awkward attempts to discuss sometimes uncomfortable topics to making plans for integrating reflection into their team’s meetings has been incredibly rewarding. And now we have the data to show that the intervention is effective, valuable, and useful for teams.

As CTSAs turn their focus to Translational Science, improving how teams conduct CTR becomes increasingly important. Yet the SciTS field currently has few evidence-based interventions that Team Science leaders and CTRTs themselves can implement easily and confidently. It is our hope that the work presented here will encourage more intervention developers to create accessible, low-barrier interventions that move the needle on teamwork. Without such interventions, CTRTs will continue to struggle in creating and implementing efficient, effective team processes, hindering their ability to do the high-impact, field-changing research the world needs. With them, CTRTs can increase their likelihood of advancing new treatments or process interventions into practice in support of improving human health.

## Supporting information

10.1017/cts.2025.10161.sm001 Rolland et al. supplementary material Rolland et al. supplementary material

## Supplementary material

The supplementary material for this article can be found at https://doi.org/10.1017/cts.2025.10161.
