# Supplementary material for: Optimizing an evidence-based team-building intervention for dissemination: Collaboration Planning 2.0
Source: J Clin Transl Sci. 2025 Sep 23;9(1):e234. doi: 10.1017/cts.2025.10161 (PMC12695499; doi:10.1017/cts.2025.10161)
Supplement: Rolland et al. supplementary material [file S2059866125101611sup001.docx]

**Supplementary Material**

**Appendix A: Collaboration Planning Session Worksheet**

**Project Title**:

**Team Members:**


**Session Date:**

**Facilitator:**

| **Question** | **Team Notes** |
| --- | --- |
| **Section 1: Team Vision** | |
| 1.A. What is the overarching research question or problem you’d like to answer/solve with this particular project? |  |
| 1.B. What is the longer-term vision for this team? |  |
| 1.C. For this particular project, what does success look like in tangible terms? (e.g., collect preliminary data on X, pilot test an intervention) |  |
| 1.D. How can your team create a shared vision of what success looks like for the project as a whole and the individual components? (e.g., kick-off meetings, highlighting the vision at each meeting) |  |
| **Section 2: People, Roles, & Responsibilities** | |
| 2.A. Who is on this team and what skill set do they contribute to achieving your team’s shared goals for this project? |  |
| 2.B. Now that the project is about to launch, are there skill sets that are missing from your original team? |  |
| 2.C. Do you have early-career researchers on the team that may need guidance on explaining/annotating their role on this project for their promotion package? |  |
| **Section 3: Team Outputs** | |
| 3.A. What kinds of outputs do you anticipate arising from this project? Please specify what and where, if applicable. |  |
| 3.A.i. Publications |  |
| 3.A.ii. Intellectual property/patents |  |
| 3.A.iii. Data sets |  |
| 3.A.iv. Other scholarly output (conference talks, posters, etc.) |  |
| 3.A.v. Public outreach/communication (report back of results to communities, media, etc.) |  |
| 3.A.vi Preliminary data for future funding proposals |  |
| 3.B. What will your authorship or attribution policies be? |  |
| **Section 4: Team Culture** | |
| 4.A. What are some of your team norms and expectations? How would you describe your team’s culture to a new person? (e.g., we value autonomy and collaboration equally; when something doesn’t go as planned, we regroup and decide next steps together; we set aside time for getting to know each other and have fun; we believe good ideas and leadership can come from anywhere on the team) |  |
| 4.B. How can you make that team culture explicit and communicate and enforce those team norms and expectations for both existing and new team members? |  |
| 4.C. How will your team make sure all voices are heard, that all members are encouraged to participate and add their unique perspectives to the conversation? |  |
| **Section 5: Team Processes & Team Functioning** | |
| 5.A. What is your process for making decisions about: |  |
| 5.A.i. Scientific direction? |  |
| 5.A.ii. Resource allocation? |  |
| 5.A.iii. Personnel? |  |
| 5.A.iv. Other? |  |
| 5.B. What is your process for resolving disputes such as those over resources or deliverables? Consider different kinds of conflict, including Interpersonal conflict vs scientific conflict and conflicts among staff vs conflicts between PIs. |  |
| 5.C. How can your team assess if it is functioning well? What are some of the red flags for a poorly functioning team and signs of a highly functioning team? |  |
| **Section 6: Project Management & Infrastructure** | |
| 6.A. Project Management: How do you anticipate managing the project? |  |
| 6.A.i. Is there a designated project manager? |  |
| 6.A.ii. How will tasks be identified, assigned, tracked, and judged complete? |  |
| 6.A.iii. Who will organize meetings and record the discussion and decisions? |  |
| 6.A.iv. How often will your team meet and by what modality (in-person, WebEx, phone)? |  |
| 6.A.v. Are there sub-teams that will meet? If so, how will the outcomes of those meetings be communicated to the larger group? |  |
| 6.B. Project Infrastructure and Shared Tools: |  |
| 6.B.i. What communication technologies (WebEx, email) will you use to work together? |  |
| 6.B.ii. What coordination technologies (shared calendar, Box, shared drive, project management tools) will you use to work together? |  |
| 6.B.iii. Are there outside collaborators who will need access to UW systems? If your collaborators are at other institutions, will the UW resources you’re using be accessible to the entire team? |  |
| 6.C. Information Management: |  |
| 6.C.i. How are the results of meetings and communications documented and stored so they are accessible to the team and for future use? |  |
| 6.C.ii. How will you document where each type of information (e.g., meeting notes, SOPs, forms, tasks, team contact lists) lives and train everyone on the team so they know what information goes into which tool and where to find different kinds of information? |  |
| 6.D. Data Management: |  |
| 6.D.i. At a high level, how will data be managed? |  |
| 6.D.ii. What will your data sharing policy be? Who has access to the raw data or other data sets from the project? |  |
| 6.D.iii. Do you need any data use agreements with partners external to the lead team’s institution? |  |
| **Section 7: Implementation & Maintenance of the Collaboration Plan** | |
| 7.A. The next step in the Collaboration Planning process is to take today’s discussion and create a Collaboration Plan. This plan can take whatever form you think will be most useful for your team and should be made available to all current and future team members. To help you decide on the form your plan should take, please consider the following questions: |  |
| 7.A.i. How can you see your team using this Collaboration Plan? |  |
| 7.A.ii. What format would be useful for your team? (E.g., a wiki page, an editable shared document, a contract that everyone signs when joining the team) |  |
| 7.A.iii. How can your team work together to create that Collaboration Plan? |  |
| 7.B. How can you build in reflection time at each project milestone to assess your team processes and your alignment with the Collaboration Plan? |  |
| 7.C. How can you allocate resources (e.g., time, effort) to support team function? |  |
| 7.C.i. Can you add a short “team function” question to each leadership and/or team meeting? Examples:  1. Did our team work as effectively as possible this past [month, week]?  2. What did our team learn this week and how does that impact what we do next?  3. What is one thing that happened this month that exemplified our team values?  4. How did we do this month in making progress toward our goals?  5. Where are we struggling to meet our team expectations?  6. Is there a way that our team can better support you in your work? |  |
| 7.C.ii. How can the leadership team assess team functioning? |  |

This work is licensed under a Creative Commons Attribution-NonCommercial-ShareAlike 4.0 International License.

Betsy Rolland, PhD MLIS MPH, [betsyrolland@gmail.com](mailto:betsyrolland@gmail.com)

Betsy Rolland LLC, dba The Team Science Lab

V10, updated October 17, 2023

***Please maintain this licensing statement on any adaptations you make.***

**Appendix B: Collaboration Planning Evaluation Forms**

**Collaboration Planning Intake & Scheduling Forms**

Thank you for your interest in holding a Collaboration Planning session for your team. The information below will help us develop a more customized session for your team.

1. Name
2. Email Address
3. How would you describe your role on the team (i.e., PI, CO-PI, Research Coordinator, Trainee):
4. Title and brief description of project:
5. Which of the following statements describe your team (select all that apply):
   1. Members on our team have NOT collaborated previously
   2. Members of our team HAVE collaborated previously
   3. Pre/Post Doctoral Trainees are part of our team
   4. We are an interdisciplinary team (i.e. involving two or more of the following: basic science, clinical research, population science/public health) *[You may need to update based on your disciplinary mix.]*
   5. Our team involves multiple lab groups or institutions
   6. We are working with community partners
6. Which category best describes your team's research and development activity?
   1. Generating preliminary data for pilot proposal submission
   2. Pilot awarded, launching pilot project
   3. Preparing and submitting applications for extramural funding award
   4. Extramural Funding awarded, launching extramural project or center
   5. Other
7. Please list the names and email addresses of team members you would like to invite to the Collaboration Planning session.
   1. NOTE: There isn't a limit on the number of attendees per session. We encourage you to involve as many of your collaborators/team members as possible to ensure a robust discussion of team processes!
8. Do you have a regularly scheduled team meeting slot that could potentially be used for this session? If so, please enter the time/day you generally meet.
9. Does your team have any particular challenges or concerns you’d to focus on during the Collaboration Planning session?

**Collaboration Planning Pre-survey**

Thanks for registering to participate in a Collaboration Planning session. Please take a moment to fill out this brief survey before your session. The demographic data will be used to understand the diversity of translational teams, but please note your responses are confidential and all data will be reported in aggregate with any identifying information removed.

If you have any questions, please contact us at *[email address]*.

1. Please rate your agreement with the following phrases describing your team's collaboration practices: (strongly disagree, disagree, neutral, agree, strongly agree)
   1. I can describe the research vision of this team
   2. I know who is on this team and what they contribute to the research vision.
   3. I understand my role on this team
   4. I understand the role of other members of this team
   5. I can describe our team’s planned outputs (deliverables)
   6. Our team has clearly defined authorship or attribution policies
   7. I understand the communication norms, culture, and expectations of our team.
   8. I understand our team’s approach to managing conflict.
   9. I understand how project management will be handled in this project.
   10. I can access the information and data I need to complete my role on this project
   11. Our team has a plan to reflect on both our scientific progress and our team work
2. What are your goals for participating in this Collaboration Planning session?

**Collaboration Planning Evaluation (Post-Session) Survey**

Thanks for participating in Collaboration Planning! Please take a moment to fill out this brief survey after your session.

If you have any questions, please contact us at *[email address]*.

1. Which sections of the Collaboration Planning (CP) worksheet did your team discuss during your session? [check all that apply.]
   1. Section 1: Team Vision
   2. Section 2: People, Roles, & Responsibilities
   3. Section 3: Team Outputs
   4. Section 4: Team Culture
   5. Section 5: Team Processes & Team Functioning
   6. Section 6: Project Management & Infrastructure
   7. Section 7: Implementation & Maintenance of the Collaboration Plan
2. Which sections of the CP Worksheet do you think will have the most impact on your team's collaboration practices? [Please select your top 2-3.]
   1. Section 1: Team Vision
   2. Section 2: People, Roles, & Responsibilities
   3. Section 3: Team Outputs
   4. Section 4: Team Culture
   5. Section 5: Team Processes & Team Functioning
   6. Section 6: Project Management & Infrastructure
   7. Section 7: Implementation & Maintenance of the Collaboration Plan
3. Please rate your agreement with the following phrases describing your team's collaboration practices: (strongly disagree, disagree, neutral, agree, strongly agree)
   1. I can describe the research vision of this team
   2. I know who is on this team and what they contribute to the research vision.
   3. I understand my role on this team
   4. I understand the role of other members of this team
   5. I can describe our team’s planned outputs (deliverables)
   6. Our team has clearly defined authorship or attribution policies
   7. I understand the communication norms, culture, and expectations of our team.
   8. I understand our team’s approach to managing conflict.
   9. I understand how project management will be handled in this project.
   10. I can access the information and data I need to complete my role on this project
   11. Our team has a plan to reflect on both our scientific progress and our team work
4. Overall, I found this collaboration planning session to be a valuable experience:
   1. Strongly disagree
   2. Disagree
   3. Neutral
   4. Agree
   5. Strongly agree
5. What is one action you will take as a result of participating in this session?
6. Would you recommend Collaboration Planning to a colleague?
   1. Very unlikely
   2. Unlikely
   3. Undecided
   4. Likely
   5. Very likely
7. Overall, how effective was the facilitator in guiding discussion during your research Collaboration Planning (CP) session?
   1. Very ineffective
   2. Ineffective
   3. Neither effective nor ineffective
   4. Effective
   5. Very effective
8. What suggestions do you have for the session facilitator for improving the collaboration planning service? Are there things you would like to see changed or added? If so, what?

**Supplementary Material**

| **Team** | **# of team members invited to participate in a CP session (pre- and post-session survey recipients)** | **# of team members who attended CP session** | **# (%) of team members who completed pre-session survey** | **# (%) of team members who completed post-session survey** | **# (%) of team members who completed both pre- and post-session surveys** |
| --- | --- | --- | --- | --- | --- |
| 1 | 3 | 3 | 3 (100%) | 3 (100%) | 3 (100%) |
| 2 | 6 | 6 | 6 (100%) | 5 (83%) | 5 (83%) |
| 3 | 6 | 6 | 5 (83%) | 5 (83%) | 5 (83%) |
| 4 | 5 | 5 | 5 (100%) | 2 (40%) | 2 (40%) |
| 5 | 5 | 5 | 4 (80%) | 4 (80%) | 4 (80%) |
| 6 | 5 | 5 | 5 (100%) | 5 (100%) | 5 (100%) |
| 7 | 5 | 5 | 3 (60%) | 2 (40%) | 2 (40%) |
| 8 | 5 | 5 | 4 (80%) | 4 (80%) | 4 (80%) |
| 9 | 8 | 8 | 6 (75%) | 5 (63%) | 5 (63%) |
| 10 | 17 | 9 | 6 (35%) | 5 (29%) | 2 (12%) |
| 11 | 12 | 11 | 6 (50%) | 5 (42%) | 4 (33%) |
| **Total** | **77** | **68** | **53 (69%)** | **45 (58%)** | **41 (53%)** |

**Table S.1:** Participation by team. [CP=Collaboration Planning]

| **Collaboration History (could check multiple) N=9 completed registration surveys** | **# (%)** |
| --- | --- |
| Members on our team have NOT collaborated previously | 7 (64%) |
| Members of our team HAVE collaborated previously | 7 (64%) |
| Pre/Post Doctoral Trainees are part of our team | 5 (45%) |
| We are an interdisciplinary team (i.e. involving two or more of the following: basic science, clinical research, population science/public health) | 8 (73%) |
| Our team involves multiple lab groups or institutions | 4 (36%) |
| We are working with community partners | 6 (55%) |

**Table S.2:** Team Collaboration History

| **Post-session survey question** | **Average score**  **(N=45)** | **Combined positive + very positive** |
| --- | --- | --- |
| Overall, I found this collaboration planning session to be a valuable experience. | 4.4 | 41 (91%) |
| Would you recommend Collaboration Planning to a colleague? | 4.5 | 41 (91%) |
| Overall, how effective was the facilitator in guiding discussion during your research Collaboration Planning session? | 4.6 | 44 (98%) |

**Table S.3:** Participant Satisfaction Scores. *Note, scores ranged from 1-5, with 5 being the most positive response for each item (i.e. “Strongly agree”, “Very likely”, and “Very effective”)*

| Survey item* | **1** | **2** | **3** | **4** | **5** | **6** | **7** | **8** | **9** | **10** | **11** |  |
| --- | --- | --- | --- | --- | --- | --- | --- | --- | --- | --- | --- | --- |
| **Avg pre-session score** | 4.32 | 4.24 | 4.34 | 4.22 | 4.12 | 3.54 | 4.00 | 3.59 | 3.80 | 4.07 | 3.73 |  |
| **Avg post-session score** | 4.66 | 4.66 | 4.56 | 4.56 | 4.41 | 3.61 | 4.41 | 4.02 | 4.32 | 4.29 | 4.22 |  |
| **Diff**  **(post-pre)** | 0.34 | 0.41 | 0.22 | 0.34 | 0.29 | 0.07 | 0.41 | 0.44 | 0.51 | 0.22 | 0.49 |  |
|  | | | | | | | | | | | | |
| **# (%) of participants who increased score** | 12 (29%) | 14  (34%) | 9  (22%) | 13  (32%) | 12 (29%) | 13 (32%) | 12 (29%) | 13 (32%) | 18 (44%) | 13  (32%) | 16 (39%) |  |
| **# (%) of participants who decreased score** | 3  (7%) | 2  (5%) | 3  (7%) | 4  (10%) | 4  (10%) | 12 (29%) | 3  (7%) | 4  (10%) | 2  (5%) | 5  (12%) | 5  (12%) |  |
| **# (%) of participants who did not change score** | 26 (63%) | 25 (61%) | 29  (71%) | 24  (59%) | 25 (61%) | 16 (39%) | 26 (63%) | 24 (59%) | 21 (51%) | 23  (56%) | 20 (49%) |  |

**Table S.4:** Average scores on pre- and post-session surveys. N=41, *Scale : [1=Strongly disagree; 2=Disagree; 3=Neutral;4=Agree; 5=Strongly agree].*

| Survey item* | **1** | **2** | **3** | **4** | **5** | **6** | **7** | **8** | **9** | **10** | **11** |
| --- | --- | --- | --- | --- | --- | --- | --- | --- | --- | --- | --- |
| % | 93 | 88 | 95 | 88 | 90 | 51 | 78 | 56 | 73 | 81 | 66 |

**Table S.5:** Proportion of participants reporting “Agree” or “Strongly agree” at baseline for team process questions.

**Note*. Key:

1. I can describe the research vision of this team.
2. I know who is on this team and what they contribute to the research vision.
3. I understand my role on this team.
4. I understand the role of other members of this team.
5. I can describe our team's planned outputs (deliverables).
6. Our team has clearly defined authorship or attribution policies.
7. I understand the communication norms, culture, and expectations of our team.
8. I understand our team's approach to managing conflict.
9. I understand how project management will be handled in this project.
10. I can access the information and data I need to complete my role on this project.
11. Our team has a plan to reflect on both our scientific progress and our team work.
